# A comprehensive review of production, applications, and the path to a sustainable energy future with hydrogen

**DOI:** 10.1039/d4ra04559a

**Published:** 2024-08-22

**Authors:** Abdulrahman bin Jumah

**Affiliations:** a Chemical Engineering Department, College of Engineering, King Saud University P.O. Box 800 Riyadh 11421 Saudi Arabia abinjumah@ksu.edu.sa

## Abstract

Green hydrogen, a versatile and sustainable energy carrier, has garnered increasing attention as a critical element in the global transition to a low-carbon economy. This review article comprehensively examines the production, applications, and potential of green hydrogen, accompanied by the challenges and future prospects associated with its widespread adoption. The production of green hydrogen is a central focus, due to its environmental benefits and distinctive characteristics. The article delves into the various techniques and technologies employed in green hydrogen production, emphasizing the need for cost reduction and increased scale for economic viability. Focusing particularly on applications, the review discusses the diverse sectors where green hydrogen demonstrates immense promise. Challenges and limitations are explored, including the intermittent nature of renewable energy sources, high production costs, and the need for extensive hydrogen infrastructure. The article also highlights the pressing need for innovation in electrolysis technology and materials, emphasizing the potential for cost reduction and increased efficiency. As industries gradually transition to green hydrogen as a cleaner feedstock, its demand and cost-competitiveness are projected to increase. This review article thoroughly evaluates the current status of green hydrogen and provides valuable insights into its potential role in the transition to a sustainable energy system.

## Abstract 2

Green hydrogen, a versatile and sustainable energy carrier, has garnered increasing attention as a critical element in the global transition to a low-carbon economy.

## Graphical abstract

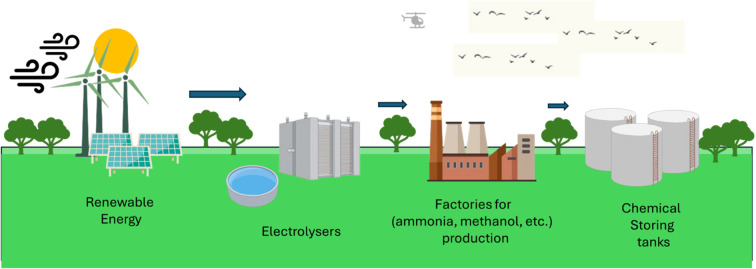

## 1. Introduction

Energy plays a pivotal role in numerous everyday functions, encompassing transportation, mobility, culinary activities, water purification, communication, and more.^1,2^ Our planet is confronted with two significant dilemmas – environmental pollution and an energy crisis, both of which have been intensified by the swift progression of human civilization.^3,4^ The growing use of fossil fuels to satisfy our current energy requirements has raised concerns about an impending energy crisis. This, in turn, has sparked a renewed enthusiasm for advancing renewable alternatives to address the increasing energy demands of the developing world.^5,6^ The overreliance on fossil fuels has induced global warming through carbon dioxide emissions, underscoring the urgent need for the enthusiastic encouragement of clean and renewable energy sources.^7^ In 2019, the annual global carbon dioxide emissions resulting from energy sources amounted to 33.3 metric gigatons, and this growth rate is projected to cause a significant rise in Earth's temperature by several degrees unless effective mitigation measures are implemented.^8^ Nowadays, an increasing number of nations are declaring their shift towards greener energy sources and more environmentally conscious forms of transportation. This phenomenon, often denoted to as the “worldwide energy transition” is gaining substantial momentum at a rapid pace.^9,10^ Some of these major transitions' projects can be found in Table 1.

**Table 1:** Illustrations of some of the most extensive renewable energy endeavors employing various technologies across the globe

Type	Name	Country	Power capacity (MW)	Year	Ref.
Biomass power plant	Alholmens Kraft power plant	Finland	240	2002	11
Hydroelectric power	Three Gorges dam	China	22 500	2003	12
On-shore wind farm	Gansu wind farm	China	7965	2009	13
Biomass power plant	Ironbridge power plant	United Kingdom	740	2012	14
Biomass power plant	Polaniec biomass power plant	Poland	220	2012	15
Photovoltaics & hydroelectric power	Longyangxia dam solar park	China	2130	2015	16
Parabolic trough and solar power tower (CSP)	Ouarzazate solar power station	Morocco	580	2016	17
Photovoltaics	Bhadla solar park	India	2245	2018	18
Photovoltaics	Huanghe hydropower hainan solar park	China	2200	2020	19

Taking proactive measures against climate change, as indicated by the International Energy Agency (IEA) study, could bring about $26 trillion in economic benefits and 65 million new jobs by 2030.^20^ Hence, renewable energy sources are increasingly gaining traction to achieve environmentally friendly and sustainable energy systems. This is due to their non-carbon, widespread availability, and high-energy-density characteristics.^21–23^ Broadly speaking, the utilization of renewable energy stands as the most appealing approach, holding the potential to substantially reduce or even eliminate the reliance on fossil fuels. Renewable energy sources have experienced a significant increase in generation and adoption over the past decade. Some of these sources are even harnessed for large-scale electricity production, as seen in the case of solar energy,^24,25^ wind energy,^26–28^ biomass energy,^29,30^ and ocean energy.^31,32^

Hydrogen (H_2_), a gas that is both colorless and odorless, possesses remarkable flammability. Several sources, such as biomass, natural gas, and water, can be used to obtain hydrogen, which is the lightest and most abundant element in the universe.^33–35^ Utilizing H_2_ as a fuel source involves the transformation of this gas into electricity within a hydrogen fuel cell (FC). These cells are distinguished by their exceptional efficiency, boasting rates of up to 60%. Moreover, they demonstrate an environmentally friendly character, as they generate no detrimental emissions, yielding only water and heat as by-products.^36,37^ H_2_ stands as an eco-friendly fuel, emitting no environmentally harmful molecules during combustion or oxidation at lower temperatures.^38,39^ H_2_ shows significant potential for reducing carbon emissions in the energy sector and achieving net-zero production by 2050. Driven by these compelling attributes of hydrogen, several nations have just unveiled plans and initiatives aimed at establishing sustainable, renewable (green) hydrogen ecosystems.^40–42^

**Fig. 1:**
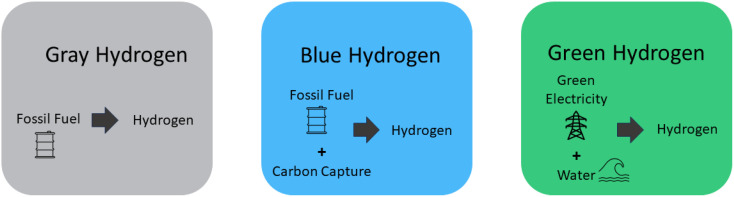
Types of H_2_ and their origin.

H_2_ can be generated from both sustainable and non-sustainable origins, resulting in the categorizations of green, blue, and gray hydrogen.^43^ These types and their origins are shown in brief in (Fig. 1).

The production of H_2_ through the utilization of fossil fuels is classified as gray H_2_, denoting its association with environmental consequences and carbon emissions resulting from the combustion of these finite resources.^44^ The majority of present-day H_2_ production stems from fossil fuels incorporating no carbon dioxide capture. While these stands as the most direct approach to hydrogen generation, its sustainability is questionable.^45^ Gray H_2_ is acquired through processes that yield greenhouse gas emissions exceeding 36.4 grams of carbon dioxide per megajoule (MJ), regardless of whether these processes rely on renewable or non-renewable resources.^46,47^ In the current landscape, the foremost origins of the H_2_ supply can be attributed to the exploitation of coal and natural gas. The industrial utilization of H_2_ spans across the globe; however, the act of producing H_2_ presents a notable concern due to the substantial carbon dioxide emissions it contributes annually. This interplay underscores the delicate balance between the practical applications of H_2_ and the environmental ramifications inherent in its generation.^48,49^ In summary, the production of gray H_2_ from fossil fuels carries significant environmental implications and carbon emissions. While fossil fuels dominate current H_2_ production, their sustainability remains uncertain.

The outcome of this process is blue H_2_, which emerges from the utilization of fossil fuels in combination with methods involving carbon utilization, storage, and absorption.^50,51^ Blue H_2_ is commonly synthesized from natural gas, frequently employing steam reforming techniques coupled with carbon capture and storage. While certain approaches to blue H_2_ production involve carbon absorption, it's important to note that this method doesn't inherently eliminate carbon emissions.^52^ Producing a substantial quantity of blue H_2_ could play a vital role in supporting the expanding worldwide and local H_2_ supply chains and their associated fuels. The highest projected efficiency for carbon dioxide absorption stands between 85% to 95%, resulting in a leakage of around 5% to 15% of the total carbon.^53^

H_2_ derived from sustainable and renewable resources is classified as green H_2_, signifying its origin through environmentally friendly methods that harness sources like solar, wind, or hydropower.^54–56^ An increasingly prominent technique within this sector, garnering noteworthy focus in recent times, is the electrolytic generation of H_2_.^57^ The production of green H_2_ using renewable energy sources is expected to increase rapidly in the near future. Multiple ongoing and forthcoming initiatives are aligned with this trajectory.^58,59^ Nonetheless, achieving substantial cost reduction necessitates increased mass production, dedicated research, and comprehensive development efforts. In accordance with this pattern, the scope of projects has experienced exponential expansion in recent times. H_2_ generated from renewable sources has the hypothetical to greatly enhance renewable energy output. It is currently technically viable and has the imminent potential to become a prominent global economic contender.^60^ Anticipations from experts indicate that, by 2050, the cost of green H_2_ will probably fall to a level below $1 per kilogram, thus rendering green H_2_ a more competitive option. This underscores the pressing requirement for persistent research and development efforts in the realm of H_2_ energy. Such investments are essential because H_2_ is forecasted to emerge as the preferred choice for fuel in the forthcoming years, primarily due to its substantial energy content and environmentally advantageous attributes.^61^

## 2. Production methods

Despite its prevalence in the universe, H_2_ is mostly found in combination with other elements on Earth. Thus, the production of H_2_ hinges on its extraction from various compounds. H_2_ can be produced from various sources, containing sustainable resources like biomass and water, as well as non-renewable sources such as crude oil, coal, and natural gas. Although fossil fuels are a cheap way to produce H_2_, concerns about their limited reserves and environmental impact have steered the search for alternative methods.

Some of the already used methods, such as pyrolysis, have several advantages, including the production of H_2_-rich fuel, rapid and efficient decomposition of feedstock, and great flexibility. However, there are also certain disadvantages, such as the high energy requirements and the potential for tar formation. Gasification offers several advantages, including the capability to convert a wide range of feedstocks, extraordinary productivity, and the potential to generate value-added products alongside H_2_ production. Nevertheless, there are also some disadvantages, such as electrode deactivation, substantial energy requirements, and the necessity for exceptionally durable equipment. Reforming offers several advantages, including a high capacity to reform diverse materials, cost-effective construction, rapid response capabilities, and compactness. However, there are also some drawbacks, such as high energy requirements, a significant reduction in electrode lifetime, and the need for catalyst regeneration. The data included in Table 2.

**Table 2:** A comparison between pyrolysis, gasification, and reforming in the production of green hydrogen

Type	Drawbacks	Benefits	Ref.
Pyrolysis	High required energy	High flexibility	62–64
		Fast and efficient feedstock decomposition	
	Possibility of tar formation	H_2_-rich fuel production	
Gasification	Need for high resistant equipment	High productivity	65–67
	High required energy	Ability to convert a variety of feedstocks	
	Deactivation of electrodes	High potential to produce value-added products along with H_2_	
Reforming	Considerable reduction in electrode lifetime	High-speed responding quality	68–70
	Need for catalyst regeneration	Cheap construction costs	
	High energy requirement	High capacity to reform varying materials	
		Compactness	

### 2.1. Steam methane reforming

**Fig. 2:**
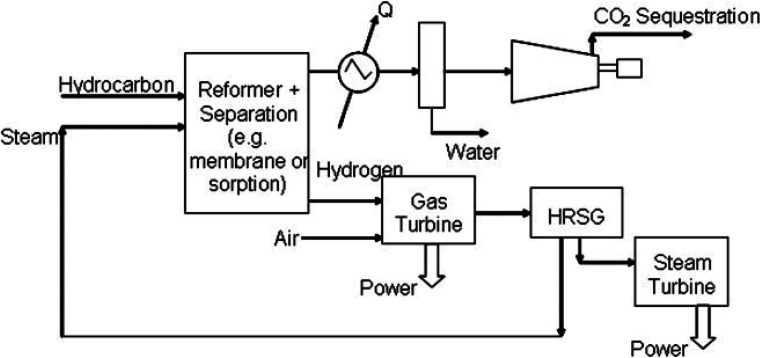
Representation of the SMR process.^71^

Steam methane reforming (SMR) (Fig. 2) is the most widely used technique for producing H_2_ from natural gas.^72^ In a typical SMR process, steam is combined with natural gas and methane-rich gases (like those from landfills and biogas) using a catalyst to construct hydrogen and carbon monoxide (CH_4_ + H_2_O → CO + 3H_2_). Typically, SMR yields a H_2_-rich gas composition, containing approximately 70% to 75% H_2_ by dry mass, along with small quantities of carbon dioxide, carbon monoxide, and methane. Reforming natural gas accounts for about 50% of the global H_2_ supply currently.^73^ Nonetheless, given that natural gas is consumed directly and results in the production of CO_2_, this method raises significant environmental concerns.^74^ A much greener approach is imperative for the future.

### 2.2. Plasma

The introduction of innovative technologies, such as plasma, within thermochemical conversion methods, offers new avenues for the cost-effective production of H_2_, accompanied by the creation of value-added products. Plasma is categorized into two main types: non-thermal and thermal plasma, distinguished by their characteristics, including energy levels, temperature, and electronic density.

**Fig. 3:**
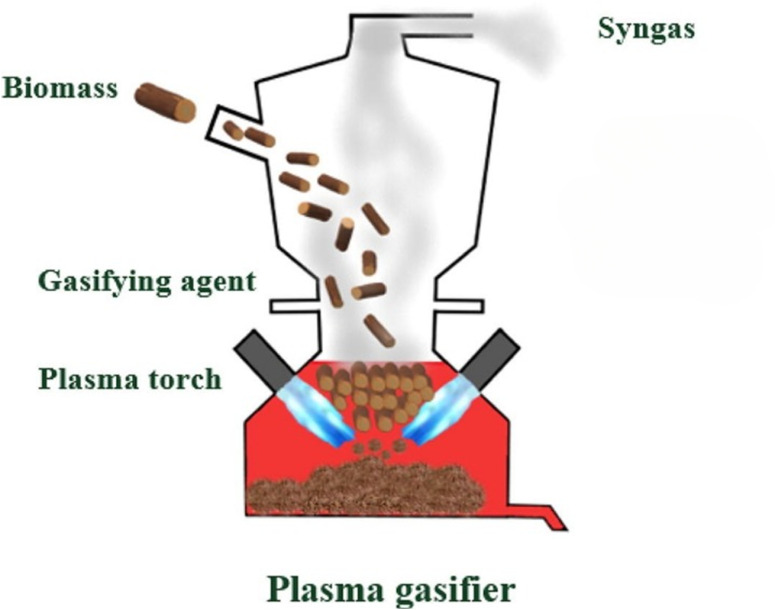
Representation of the production of green H_2_ with plasma route.^75^

Plasma technologies work by energizing gas streams through electrical discharge (Fig. 3). This process results in the generation of various components, including positively charged ions, negatively charged electrons, neutrals, reactive and excited species, an electromagnetic field, and photons. In conditions that deviate from normal atmospheric pressure and temperature, these phenomena facilitate the efficient conversion of biomass into H_2_ through oxidation.^76,77^ Conversion methods utilizing plasma technology hold promise for the production of valuable chemicals in addition to H_2_, and their generation of harmful pollutants is virtually negligible.^78,79^ Furthermore, by integrating with complementary processes, these methods can yield a H_2_ product of exceptionally high purity.^80^

Kuo and colleagues^75^ conducted a comprehensive analysis by a DC plasma torch reactor to evaluate the suitability of various biomass feedstocks for H_2_ production. Their investigation encompassed a diverse range of sources, including pine wood chips, grape marc, forest residues, rice straw, and macroalgae. The chief aim of their research was to discern how the choice of biomass influenced not only H_2_ production but also the formation of harmful compounds and the overall gasification yield achieved through plasma technology. Notably, the outcomes revealed a consistent H_2_ concentration of 68 mol% in the syngas generated from all the studied biomass sources. Non-woody biomass sources showed a higher presence of sulfur compounds compared to woody sources, which can be explained by the inherent traits of non-woody materials. Among the tested options, pine wood emerged as the most favorable choice due to its exceptional efficiency in plasma gasification and the minimal presence of impurities in the resulting syngas, underlining its potential for sustainable H_2_ production.

Wu and colleagues^81^ conducted a comprehensive investigation into the presentation of methanol decomposition through a unique liquid-phase discharge setup, designed for enhanced visualization. The use of a high-speed camera in their study gave a detailed understanding of methanol decomposition and liquid-phase discharge processes. By changing the electrode spacing, the researchers were able to create two different plasma discharge modes: discharge (GD) and gliding arc discharge (GAD) glow. GD's current and voltage curves closely resemble the sinusoidal waveform of AC power supplies, with a discharge power range of 130.4 to 460.2. In contrast, GAD exhibited a unique feature of bipolar pulses, characterized by high transient peak currents (ranging from 420.6 to 690.9 mA), resulting in a lower discharge power of 30.7–110.3 W. Initial analyses revealed that GAD's energy consumption was notably lower than that of GD, primarily because of disparities in their discharge characteristics. By optimizing the process, we achieved an energy consumption rate of 1.63 kW h per cubic meter of H_2_ for hydrogen production. The result of this approach was a gaseous product with a maximum hydrogen proportion of 63.21%, and carbon monoxide as the primary byproduct at 26.38%. These findings shed light on an efficient and sustainable method for hydrogen production.

Tabu and their team^82^ accomplished the development of low-temperature, atmospheric pressure plasma reactors utilizing the principles of gliding arc (glidarc) discharges and transferred arc (transarc). These reactors were meticulously designed, constructed, and meticulously characterized to facilitate the conversion of low-density polyethylene (LDPE), serving as a representative model for plastic waste, into H_2_. Their experimental findings revealed a clear relationship between voltage levels and H_2_ production rates and efficiency in both reactors. As voltage levels increased, H_2_ production exhibited a steady rise. The transarc reactor achieved a maximum H_2_ manufacture of 0.33 mmol g^−1^ LDPE, while the glidarc reactor surpassed this with a peak hydrogen production of 0.42 mmol g^−1^ LDPE. The transarc reactor showed increased hydrogen production with a narrower electrode-feedstock spacing. However, the glidarc reactor exhibited greater hydrogen generation when flow rates were moderate. Remarkably, despite their significantly different operational modes, both reactors delivered comparable H_2_ production results. These findings represent a substantial step forward in the utilization of plastic waste for H_2_ generation, offering valuable insights into the effectiveness of the transarc and glidarc technologies.

Conventional approaches to H_2_ production encompass processes like water electrolysis, biomass gasification, coal gasification, and steam methane reforming. However, many of these methods, particularly those relying on fossil fuels, are associated with substantial carbon emissions, which run counter to the aim of achieving carbon neutrality. Future H_2_ production should prioritize renewable resources and minimizing carbon emissions.^83–85^

### 2.3. Renewable energy-powered hydrogen generation systems

The efficient production of H_2_ through renewable energy sources necessitates a thorough examination of the optimal configurations, which are contingent on factors for example geographical location, the obtainability of energy storage solutions, the choice between on-grid or off-grid systems, and the specific water electrolysis techniques employed.

Water electrolysis, despite being naturally endothermic, needs a higher voltage than the theoretical electrolysis voltage because of ohmic and overpotential loss.

**Fig. 4:**
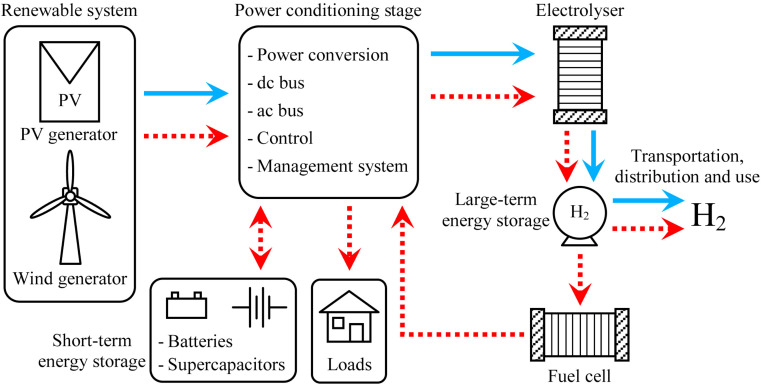
Configurations for the integration of electrolysers with renewable energies in stand-alone systems.^86^

In a study conducted by Gandia *et al.*^87^ they conducted, simulations to explore the production of H_2_ through wind energy. Their findings revealed significant temperature fluctuations during transient operation, with notable temperature spikes observed under high-power generation conditions and, conversely, temperature decreases during low-power generation scenarios. Notably, the study identified a safety concern related to gas crossover, specifically the presence of O_2_ in the H_2_ stream and H_2_ in the O_2_ stream. The crossover gases reached high concentrations, especially when the gas volume decreased due to low power generation. This was mainly because the current determined the total of hydrogen gas manufactured. This underscores the importance of addressing safety considerations in H_2_ production processes. Hence, the variability in the power supply altered the condition of the electrolyzer, impacting the purity of the gas produced.^88^ Furthermore, The research by Ursúa *et al.* (Fig. 4),^86^ they found that during the operation of an alkaline water electrolyzer without additional devices, a requirement was established to maintain a minimum power load of 40%. The objective is to maximize the employment of renewable energy resources by evaluating if it's feasible to operate alkaline water electrolyzers below the minimum power load.^86^ They aimed to improve the utilization of renewable energy sources. The outcomes of their study revealed that the system could sustain operation for up to 20 minutes under these conditions. Moreover, by implementing these adjustments, they were able to reduce the frequency of operational halts in a water electrolyzer powered by photovoltaic energy by half. Consequently, this approach led to an enhancement in energy efficiency by an additional 6.3%.

In their extensive research, Stansberry *et al.*^89^ embarked on a series of experiments employing a 60 kW proton exchange membrane (PEM) water electrolyzer driven by a combination of wind power and photovoltaic sources. Within the complex system, the most significant energy loss, marked by the inadvertent release of hydrogen gas, was observed in the pressure swing adsorption dehumidification unit, closely followed by energy losses within auxiliary equipment and the power consumption associated with alternating/direct current (AC/DC) conversion units. The overall efficiency of water electrolysis was greatly affected by the accumulation of these losses, especially at lower electric power levels, resulting in a rated current drop below 50%. These fluctuations in power delivery resulted in similar adverse scenarios for the electrolyzers. As such, it becomes paramount to improve a comprehensive sympathetic of the mechanisms underlying the varying capabilities to support such fluctuating operations. This understanding can be attained by shedding light on the factors that dictate these abilities, which encompass factors like cell structures and the integration of auxiliary equipment. These insights are essential for improving the competence of water electrolysis in renewable energy systems.

Photovoltaic and wind energy production operate in diverse time cycles and exhibit varying power output fluctuations. These traits give rise to several challenges in the operation of electrolyzers. Notably, fluctuating power can lead to electrode degradation owing to abrupt shifts in electrode potential. A significant factor in this degradation is the reverse current generated during operational halts, resulting in a substantial deterioration of electrode performance.^76^

### 2.4. Water splitting by photocatalysis

Harnessing the process of photocatalytic hydrogen production over water splitting shows promise as an environmentally friendly method for generating green H_2_, thanks to its cost-effectiveness and minimal energy requirements. The direct process of splitting water into O_2_ and H_2_ is thermodynamically challenging under standard ambient conditions, pressure off and temperature. The reaction is mainly caused by the high heat release, with a Δ*G* of 237.1 kJ mol^−1^ for water. It's worth mentioning that this reaction is not completely non-spontaneous and can be aided by catalysis.^90^ Water splitting plays a pivotal role in producing clean and renewable H_2_ for various applications, including H_2_ fuel cells and energy storage, with minimal environmental impact since it does not produce harmful greenhouse gas emissions. The equations employed to represent the process of photocatalytic H_2_ production are as provided below.^91^

1 h^+^ + □ + H_2_O → H^+^ + □ + OH

2 O_2_ + 2e^−^ + 2H^+^ → H_2_O_2_

3 O_2_ + e^−^ → O_2_^−^

4 H_2_O_2_ + O_2_^−^ → OH + OH^−^ + O_2_

5 h^+^ + □ + OH^−^ → OH

6 H^+^ + e^−^ → H

7 H + H → H_2_

**Fig. 5:**
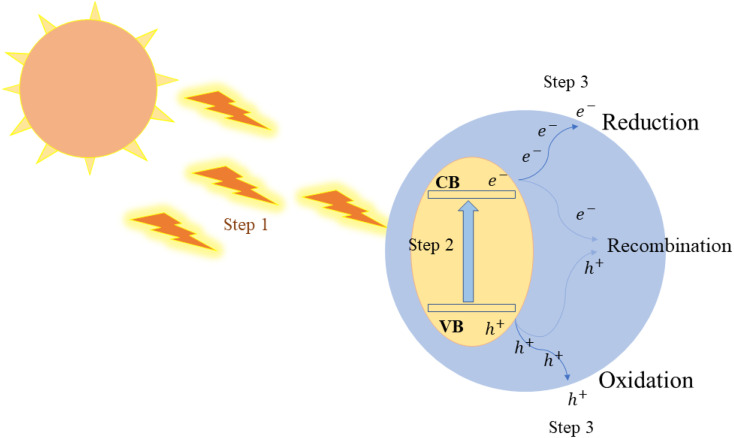
Illustration of the photocatalyst water splitting process, (1) the absorption of light radiation from a light source, (2) the separation of electron–hole pairs, and (3) redox reaction.

The foundation of photocatalytic H_2_ production lies in the semiconductor photocatalyst, which utilizes solar energy to split water. When light of a specific wavelength (a photons with a particular energy) strikes the photocatalyst, it energizes electrons from the valence band to the conduction band (CB). This event results in the creation of electron–hole (e^−^–h^+^) pairs, which play a pivotal role in driving the redox reactions occurring on the surface of the photocatalyst. Basic water splitting illustration is found in (Fig. 5).

Yan and his team,^92^ achieved a remarkable milestone by developing a novel Ni_2_P/NiS@polymeric carbon–oxygen semiconductor (PCOS). Their work resulted in a groundbreaking achievement, with a notable production rate of 70.2 μmol h^−1^ of O_2_ and 150.7 μmol h^−1^ of H_2_ produced for every 100 mg of photocatalyst. Interestingly, the reaction solution also exhibited the presence of H_2_ peroxide, initially at a rate of approximately 100 μmol h^−1^ over the first 2 hours. This hydrogen peroxide had a detrimental impact on the photocatalyst's performance. However, the introduction of MnO_2_ effectively mitigated this negative effect, resulting in excellent and stable rates of photocatalytic H_2_ and O_2_ production.

**Fig. 6:**
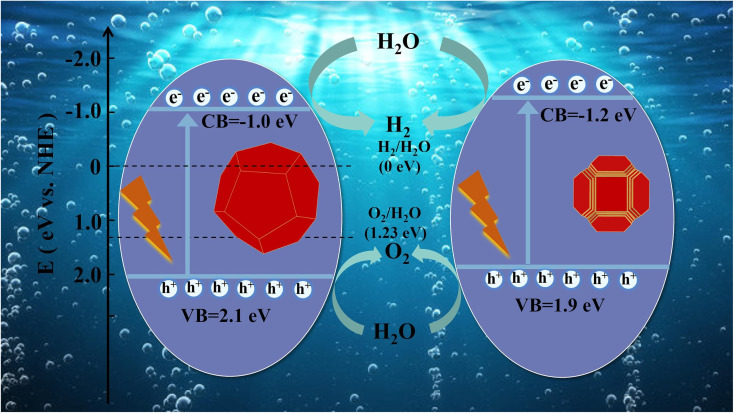
Schematic diagram of photocatalytic water splitting of samples.^93^

Ruan and colleagues (Fig. 6)^93^ introduced a groundbreaking method that marks the inaugural attempt to leverage a straightforward ethylenediaminetetraacetate (EDTA) etching process. Their goal was to enhance the number of active surface sites on photocatalysts and reduce particle size, all while preserving high crystallinity. Among the tested materials, STO-2 demonstrated remarkable performance, achieving the highest activity levels. Specifically, it facilitated H_2_ production at an impressive rate of up to 310 μmol g^−1^ h^−1^ and O_2_ evolution at 155 μmol g^−1^ h^−1^. What makes their work even more intriguing is that the EDTA etching technique holds substantial promise for broader applications. Since EDTA can interact with a wide array of metals, this uncomplicated method has the potential to be further refined for the modification of various photocatalysts, enhancing their performance in a myriad of applications.

Saleh and the research team^94^ delved into an exploration of various TiO_2_ nanocomposites enriched with two co-catalysts: Cu or Pt nanocrystals in the 3–4 nm range. These nanocomposites were synthesized through different methods, including photo-deposition, hydrothermal, and incipient wet impregnation. The results yielded a noteworthy discovery: the optimal H_2_ generation occurred with a mass filling of 0.3 wt% for both co-catalysts. What's particularly remarkable is that, even in the absence of a precious metal like Pt, the Cu/TiO_2_ nanocomposites, produced through the photo-deposition method, demonstrated a preliminary degree of 24 mmol h^−1^ g^−1^. This rate was 3.5 times greater than those synthesized using the hydrothermal method and 1.4 times greater than those produced with the impregnation method. Conversely, for Pt co-catalysts, the highest rate was observed in the impregnation-synthesized composites, clocking in at 58 mmol h^−1^ g^−1^, surpassing the rates from the photo-deposition and hydrothermal synthesis methods by 1.6 and 1.1 times, respectively.

From (Table 3). The current analysis underscores that non-green methods of hydrogen production, including SMR and coal gasification, demonstrate superior efficiency and cost-effectiveness when compared to their green counterparts, such as electrolysis powered by renewable energy. Despite their environmental drawbacks, these conventional methods offer a more mature and economically viable pathway for large-scale hydrogen production in the immediate term. However, the urgent need to mitigate climate change and reduce greenhouse gas emissions necessitates a dual focus in future research endeavors.

**Table 3:** Energy efficiency, cost and characters of hydrogen production by different methods

Production method	Energy efficiency%	Production cost, € per kg H_2_	Characters
SMR	70–85	0.56–1.12	High efficiency, low cost, mature technology, large emissions
Partial oxidation of methane	60–78	0.78–1.68	
Coal gasification	50–70	0.56–1.12	
Electrolysis of water (fossil energy)	62–82	1.79–3.36	High power consumption, high cost, high H_2_ purity
Wind electrolysis of water	—	—	Zero emissions, high cost, low conversion rate
Solar electrolysis of water	—	—	

It is imperative to enhance the cost-competitiveness and efficiency of green hydrogen production technologies. Significant advancements are required in areas such as electrolyzer technology, renewable energy integration, and novel catalyst development to bridge the gap between green and non-green hydrogen production. Furthermore, comprehensive cost-benefit analyses and life cycle assessments should be prioritized to ensure that the environmental benefits of green hydrogen are realized without compromising economic feasibility. Therefore, the next frontier in hydrogen research should aim to lower the production costs of green hydrogen while simultaneously improving its efficiency. This dual approach will not only facilitate a more sustainable hydrogen economy but also align with global environmental and economic goals. Only through such concerted efforts can we transition to a truly sustainable and scalable hydrogen infrastructure.

## 3. Storage

In the pursuit of efficient hydrogen storage solutions, researchers focus on several key material characteristics to ensure practical viability. High hydrogen storage capacity, both gravimetric and volumetric, remains paramount. Materials like metal hydrides and chemical hydrides, such as magnesium hydride (MgH_2_) and ammonia borane (NH_3_BH_3_), respectively, offer substantial storage capacities. However, challenges such as the high temperatures required for hydrogen release in metal hydrides and the often-irreversible nature of chemical hydrides under practical conditions necessitate innovative approaches. Physisorption materials, like metal–organic frameworks (MOFs) and carbon nanotubes, provide high surface areas and potential for low-temperature storage, though they typically require cryogenic conditions to achieve meaningful storage densities. Thus, achieving a balance between high capacity and operational practicality is essential.

Moreover, the kinetics of hydrogen absorption and desorption is a critical focus area, with researchers aiming to enhance these rates to facilitate rapid and efficient hydrogen storage cycles. This involves exploring nanostructured materials, which can offer increased surface areas and improved kinetics, and composite materials, which can tailor properties through synergistic interactions. Stability, both thermal and chemical, is also crucial to ensure durability over many cycles and to maintain performance without degradation. Safety and cost-effectiveness further underpin the practical deployment of these materials, necessitating that they be non-toxic, non-explosive, and economically viable for large-scale use. As such, the development of hydrogen storage materials is a multifaceted challenge, requiring a comprehensive approach to optimize capacity, kinetics, stability, and safety while maintaining economic feasibility.

The effectiveness of materials in storing H_2_ is closely tied to their physical and chemical characteristics, with a particular emphasis on their thermodynamic and kinetic properties.^95^ Up to now, the predominant technological challenge in establishing a sustainable H_2_ economy has been the creation of effective H_2_ storage systems. When evaluating methods and materials for H_2_ storage, it's essential to consider various factors, including the design of high-pressure tanks, the densities of H_2_ in terms of weight and volume, refueling speed, energy efficiency, cost, durability, adherence to standards, technical readiness, and comprehensive assessments of both life cycle and efficiency.^96^ To make H_2_ suitable for transportation, it's essential to enhance its energy density. Several methods have been suggested to achieve this, including liquefaction, compression, the formation of metal hydrides, and the utilization of liquid organic transporters such as conversion into energy carriers like methanol and ammonia.^97,98^

### 3.1. Compressed hydrogen

Compression technology provides a direct approach to H_2_ storage. Nevertheless, this approach is noticeably ineffective in terms of volume and weight, due to the low density of compressed H_2_ – around 42.2 kg per cubic meter at 69.0 MPa. Extremely high pressures, reaching up to 70.0 MPa, are required to achieve higher volumetric energy density in H_2_ fuel cell electric vehicles, necessitating the use of tanks capable of withstanding such pressures. Currently, these high-pressure tanks are crucial for achieving a driving range similar to traditional fuel-powered vehicles.^99^ Storing hydrogen in high-pressure tanks (up to 700 bar) is a mature technology. Innovations are focused on: (1) developing lightweight composite materials for tanks to improve gravimetric efficiency and safety, and (2) implementing advanced safety systems to monitor and control pressure and temperature, reducing risks of leakage and explosion.

### 3.2. Liquid hydrogen

Liquid H_2_ presents itself as a suggesting option for efficient H_2_ storage, mainly because of its exceptional purity and high density, which measures at approximately 70.8 kg of H_2_ per cubic meter. The density of this is almost 800 times higher than that of uncompressed H_2_ at standard temperature and pressure (0.08988 kg m^−3^). However, a significant drawback of H_2_ liquefaction is the need for extremely low temperatures (around −253 °C), resulting in substantial energy consumption. Liquid H_2_ becomes less feasible for long-term storage or transportation due to the cooling requirement. Another concern relates to the conversion of orthohydrogen to para-hydrogen, which generates heat through an exothermic isomerization process. The transformation of liquid H_2_ into gaseous H_2_, known as the boil-off phenomenon, happens due to heat during storage and transportation.^100^ Storing hydrogen as a liquid at −253 °C offers high energy density. Pathways for improvement include: (1) advancing cryogenic insulation materials and techniques to minimize boil-off losses, and (2) enhancing the efficiency of hydrogen liquefaction processes to reduce energy consumption.

### 3.3. Methanol

Storing H_2_ and converting carbon dioxide through hydrogenation are both advantages of methanol. The ‘power-to-product’ concept often connects methanol with the use of renewable electricity to produce chemical fuels. Methanol can be employed to generate H_2_ through processes such as steam reforming, thermolysis, and partial oxidation (POX).^101,102^ However, the utilization of methanol as a means of H_2_ storage poses environmental concerns at the point of use since it results in the release of CO_2_ either directly or during its degradation. Additionally, the process of separating and capturing CO_2_ comes with significant operational expenses and energy consumption. For instance, the conventional method of CO_2_ separation and capture, involving the use of amine solutions, demands approximately 1.1 kW h per kilogram of CO_2_.^103^

### 3.4. Formic acid

The hydrogenation of CO_2_ to form formic acid is an atom-efficient process that avoids water waste, making it a sustainable option for H_2_ storage.^104^ Additionally, formic acid exhibits promising characteristics as a H_2_ carrier. It can store approximately 4.3% of H_2_ by mass, and its high density, measured at 1.22 kg m^−3^, results in a significant volumetric density of 53 g L^−1^.^105^ When compared to alternative H_2_ storage materials, the decomposition pathway of formic acid has garnered significant interest due to its low reaction enthalpy and the fact that it can be readily generated at temperatures as low as room temperature, up to 100 °C.^106^ These features make formic acid a compelling candidate for H_2_ storage solutions, especially when considering both its efficiency and practical storage capacities. As the pursuit of efficient and sustainable H_2_ storage methods continues, formic acid stands out as a viable option with the hypothetical to diversion a fundamental part of the H_2_ economy of the future.

### 3.5. Ammonia

In essence, effective H_2_ carriers should possess a high H_2_ content, offer ease of storage and transport, and readily decompose into H_2_ when required.^107^ NH_3_ has garnered significant attention as a H_2_ carrier due to its notable attributes, including a high H_2_ content of 17.6 wt%, absence of carbon, and its ability to transition into a liquid state under mild conditions.^108,109^

Furthermore, NH_3_ can be effectively broken down into a blend of N_2_ and H_2_ gases, resulting in the generation of larger quantities of high-purity H_2_ while leaving no carbon footprint. This distinguishes it from hydrocarbon-based organic carriers like methane and methanol, which inevitably produce carbon dioxide. NH_3_ also offers the advantage of being easier to maintain in a liquid state due to its lower boiling point compared with H_2_ or methane. At room temperature, NH_3_ can be liquefied with a moderate pressure of 1.0 MPa.^110,111^

Economic assessments have demonstrated that NH_3_ holds greater promise in comparison to conventional fuels as methanol, liquefied petroleum gas (LPG), natural gas, gasoline, and hydrogen, primarily due to its absence of CO_2_ emissions.^112^ Furthermore, when compared to liquid H_2_ (8.49 MJ L^−1^) and compressed H_2_ (5.0 MJ L^−1^ at 70.0 MPa and 25 °C), liquid NH_3_ boasts a greater volumetric energy density, measuring at 10.5 MJ L^−1^.^113^ NH_3_ further distinguishes itself with a superior heat of combustion compared to liquid H_2_ at 8.58 MJ L^−1^ and nearly doubles the value of compressed H_2_ at 5.0 MJ L^−1^. Additionally, NH_3_ is less dense than air (0.769 *versus* 1.225 kg m^−3^ at standard temperature and pressure). Under atmospheric conditions, gaseous NH_3_ can disperse relatively quickly into the atmosphere, mitigating the potential explosion and fire hazards in case of accidental release. The fire risk is lower for NH_3_ than H_2_, mainly because NH_3_ has a higher auto-ignition temperature.^114^

### 3.6. Metal hydrides

A more feasible strategy is solid-state H_2_ storage based on hydrides, which capitalizes on the reversible reactions that certain metals and alloys can undergo with H_2_.^115,116^ Virtually all metallic elements have the capability to create binary compounds with H_2_, often referred to as elemental hydrides. Nevertheless, most of these compounds are not suitable for H_2_ storage due to thermodynamics, H_2_ storage capacity, or a combination of both factors.^117^ The formation of hydrides presents a significant safety advantage in comparison to storage methods involving pressurized gas and liquid H_2_, as it eliminates concerns about gas leakage. Ideally, hydrides should be capable of reversibly storing a substantial amount of H_2_ (over 6.5 wt% H) under moderate conditions (at or below 80 °C) for on-board applications.^118^ Metal hydrides, such as magnesium hydride (MgH_2_) and sodium alanate (NaAlH_4_), offer high volumetric hydrogen densities. Research should focus on: (1) incorporating alloying elements (*e.g.*, titanium or nickel) to reduce desorption temperatures and improve kinetics, and (2) developing nanoscale hydride particles to enhance surface area, reduce diffusion distances, and improve reaction rates.

#### 3.6.1. Factors affecting the choice of suitable metal hydride

Various factors influence the selection of metal hydride materials for H_2_ storage and compression. The intended application requires reversible hydride formation and decomposition within specific temperature and pressure ranges.^119^ Additionally, the material must demonstrate a significant capacity to store and release H_2_ under specific conditions. The pressure–composition–temperature (PCT) properties in H_2_ gas systems affect these characteristics when interacting with hydride-forming materials. On the pressure-composition isotherm, the width of the plateau determines the reversibility of storage capacity. The direction of the process is contingent upon the correlation between current H_2_ pressure and plateau pressure at the stated temperature. The plateau's inclination and the presence of hysteresis are important in H_2_ compression applications. They cause a significant reduction in the compression ratio,^120^ and affect the efficiency of the process within the specified temperature range.^121^

Selecting suitable metal hydride materials for high efficiency in reversible hydrogen storage and release involves considering several critical factors. First, the hydrogen storage capacity of the material is paramount; it should possess a high gravimetric (wt%) and volumetric capacity to store sufficient hydrogen. For instance, magnesium hydride (MgH_2_) offers a high hydrogen storage capacity of about 7.6 wt%. Secondly, the thermodynamics of the material, specifically the operating temperatures and pressures for hydrogen absorption (hydrogenation) and desorption (dehydrogenation), must be suitable for practical applications. A moderate enthalpy of formation is essential to balance storage capacity and ease of hydrogen release. Sodium alanate (NaAlH_4_), which operates at around 150 °C and 5 MPa, exemplifies favorable thermodynamics.

Kinetics is another crucial factor; the material should exhibit fast kinetics for both hydrogen absorption and desorption to minimize energy losses and reduce the time required for charging and discharging. For example, adding Ti-based catalysts to sodium alanate significantly improves its kinetics. Stability over multiple hydrogenation/dehydrogenation cycles is also vital, as the material must resist degradation and maintain its structural integrity and hydrogen storage capacity. LaNi_5_H_6_ is noted for its good cycling stability and moderate operating conditions.

Safety and environmental impact are also key considerations. The material should be safe to handle, with minimal risk of toxicity or flammability, and should have a low environmental impact during production, use, and disposal. Magnesium hydride, for instance, is relatively safe and environmentally benign compared to more reactive or toxic materials like lithium hydride. Cost and availability are important practical concerns; the material should be cost-effective and readily available for large-scale applications. MgH_2_, being abundant and inexpensive, is a popular choice for many applications.

Enhancements through additives and composites can significantly improve the performance of metal hydrides, enhancing their kinetics and thermodynamic properties. For example, mixing MgH_2_ with transition metal catalysts like Ti or Ni can dramatically enhance its hydrogen absorption and desorption rates. Finally, the suitability of a material depends on the specific application, whether for stationary storage, mobile applications, or portable devices. LaNi_5_H_6_, with its moderate pressure and temperature requirements, is suitable for various applications, while MgH_2_, due to its higher operational temperatures, is more apt for stationary storage.

#### 3.6.2. Magnesium hydride

There are many potential applications for MgH_2_, also referred to as magnesium hydride, as an energy carrier. It is cost-effective, widely available, and can store a lot of energy.^122^ MgH_2_ is a solid-state H_2_ storage material. It has a high bulk density of 110 grams per liter and can hold an impressive weight capacity of 7.6% H_2_ by weight. Additionally, it is known for its excellent safety and minimal environmental impact.^123^

Gao and colleagues^124^ introduced a solid-solution MAX phase TiVAlC catalyst directly into the MgH_2_ system, without the need for etching treatment, to enhance H_2_ storage performance. At 300 °C, the optimized MgH_2_-10 wt% TiVAlC composite can absorb about 4.82 wt% of H_2_ at 175 °C in 900 seconds and release around 6.00 wt% of H_2_ in 378 seconds. Impressively, even after undergoing 50 isothermal H_2_ absorption/desorption cycles, the composite exhibits exceptional cyclic stability and retains 99.6% of its capacity, which is 6.4 wt%. The abundant electron transfer at the external interfaces with MgH_2_/Mg is what gives the TiVAlC catalyst its remarkable catalytic activity. Abundant electron transfer occurs at internal interfaces (Ti_3_AlC_2_/TiVAlC) due to the presence of an impurity phase, Ti_3_AlC_2_, enhancing electron transfer and showing strong H_2_ affinity. This study is the first to explore the impact of impurity phases, which are commonly found in MAX phases, on all catalyst activity. It provides a distinct method for designing composite catalysts that enhance the hydrogen storage capabilities of MgH_2_.

Li and his team^125^ developed nanosheets of a medium-entropy alloy called CrCoNi. The addition of these nanosheets greatly boosted MgH_2_'s capacity for storing hydrogen at low temperatures. The dehydrogenation temperature of 9 wt% CrCoNi modified MgH_2_ decreased by 130 °C from 325 °C to 195 °C, surprisingly. Additionally, the composite of MgH_2_–CrCoNi discharged 4.84 wt% of hydrogen in only 5 minutes at 300 °C and absorbed 3.19 wt% of H_2_ in just 30 minutes at 100 °C (at 3.2 MPa). There was a decrease in activation energy by 45 kJ mol^−1^ for dehydrogenation, and a decrease by 55 kJ mol^−1^ for rehydrogenation. Through extensive cyclic kinetics analysis, it was discovered that the 9 wt% CrCoNi-doped MgH_2_ showed exceptional strength even subsequently 20 cycles, with a mere 0.36 wt% decrease in H_2_ capacity. The stability of CrCoNi was confirmed by XRD patterns during the cyclic reaction process. Additionally, there was a uniform dispersion of CrCoNi nanosheets on the surface of MgH_2_, resulting in numerous catalytic active sites and facile diffusion pathways with low energy barriers. Exceptional kinetic performance was achieved due to the synergistic catalysis that facilitated the rapid absorption and release of hydrogen atoms across the Mg/MgH_2_ interface.

A novel technique was developed by Zhang and the research team^126^ to boost the dehydrogenation and rehydrogenation capabilities of MgH_2_. The introduction of carbon-wrapped Ti and Co bimetallic oxide nanocages (Ti–CoO@C) made this possible. Through a precise hydrothermal method, the nanocages were synthesized and then mixed with MgH_2_ using mechanical ball milling. The hydrogen desorption was notably influenced, as MgH_2_ with 5 wt% Ti–CoO@C began desorbing hydrogen at 185.6 °C, a considerable 160.2 °C decrease compared to pure MgH_2_. Within a short span of 5 minutes, the composite released an astonishing 6.3 wt% H_2_ at 275 °C. The MgH_2_ + 5 wt% Ti–CoO@C composite exhibited a significant reduction in activation energy for H_2_ desorption/absorption, dropping from 169.19 kJ mol^−1^ and 83.61 kJ mol^−1^ for MgH_2_ to 137.76 kJ mol^−1^ and 35.17 kJ mol^−1^, respectively. Furthermore, the composite displayed exceptional stability, with no significant decline in performance observed even after 20 cycles. The catalyst's even distribution and the *in situ* formation of titanium and MgO are responsible for the remarkable hydrogen storage performance. In addition, the promoting effect of Mg_2_Co/Mg_2_CoH_5_ functioned as a H_2_ pump, thereby contributing to the improved performance. Furthermore, carbon played a vital part in catalyst nanosizing and in reducing the strength of the Mg–H bond in MgH_2_. As a result, the 5 wt% Ti–CoO@C + MgH_2_ composite exhibits outstanding hydrogen storage capabilities.

#### 3.6.3. Sodium alanate

Sodium aluminum hydride, commonly known as NaAlH_4_ and representing a class of materials known as alanates, is regarded as one of the greatest talented substances for solid-state H_2_ storage. This is primarily attributed to its impressive H_2_ storage capacity, which reaches as high as 7.5 weight percent. Beyond this notable feature, NaAlH_4_ exhibits the remarkable ability to undergo reversible H_2_ absorption and desorption processes under specific conditions.^127,128^

Ali and the research team^129^ successfully developed CoTiO_3_ through the solid-state method, and this novel material proved highly operative in ornamental the desorption behavior of NaAlH_4_ for H_2_ storage. The introduction of dissimilar weight percentages of CoTiO_3_ (ranging from 5 wt% to 20 wt%) had a profound impact. NaAlH_4_'s initial desorption temperature significantly decreased due to the inclusion of CoTiO_3_ catalysts. In the first desorption stage, the temperature decreased to about 130–160 °C, and in the second stage, it decreased to around 182–198 °C. These temperatures are much lower compared to untreated milled NaAlH_4_. The composite samples showed significantly faster desorption kinetics at 150 °C. A range of 3.0–3.7 was observed during the release of the NaAlH_4_–CoTiO_3_ composite. The activation energies for the two stages of NaAlH_4_ desorption were greatly decreased. They were lowered to 85.5 and 91.6 kJ mol^−1^, which is a reduction of 30.7 and 35.5 kJ mol^−1^ compared to untreated milled NaAlH_4_, respectively. The formation of Al–Co and Al–Ti alloys during the desorption of NaAlH_4_–CoTiO_3_ is responsible for the remarkable catalytic effect of CoTiO_3_. These discoveries create new possibilities for the advancement of efficient catalysts for NaAlH_4_, showing its potential for H_2_ storage purposes.

In a theoretical simulation by Mekky,^130^ the research explored the characteristics of pure Na_12_Al_12_H_48_, and their variations with an interstitial doping of C, H, and Ti atoms. These clusters are being considered as a talented system for H_2_ storage. The study found that, when compared to the interstitial space-doped clusters, the pure Na_12_Al_12_H_48_ clusters exhibited greater stability. The introduction of interstitial space-doped C, Ti, and H atoms into Na_12_Al_12_H_48_ did not significantly alter the lattice structure, and, notably, these atoms acted more than catalysts rather than traditional “interstitial space doping” elements. Additionally, the study found that the Na_12_Al_12_H_48_ cluster displayed greater stability, but less chemical reactivity compared to the interstitial-doped clusters. When interstitial space-doped C, H, and Ti atoms were added to Na_12_Al_12_H_48_, the lattice structure remained largely unchanged. This confirms that Ti, C, and H atoms play a catalytic role rather than simply being interstitially doped into space.

Urunkar and their team^131^ conducted a numerical analysis of a hydride reactor occupied with sodium alanate, specifically examining the absorption process within multiple tubes. They developed a mathematical model for the hydride reactor based on various governing equations and validated it using ANSYS Fluent. In general, water or oil is used in the hydride reactor to transfer heat while absorbing H_2_. The study replaced traditional heat transfer fluid with nanofluid for its better heat exchange properties. The research yielded results across several parameters, including the choice of nanoparticle material, nanoparticle concentration, H_2_ supply pressure, and the inlet temperature of the heat exchange fluid. The absorption rate of the CuO/HTF nanofluid showed significant improvement, specifically at a 5 vol% concentration, surpassing other concentrations and selected nanofluids. This improvement translated to a 14% reduction in H_2_ absorption time under specific conditions. Moreover, the CuO/HTF nanofluid with a 5 vol% concentration exhibited superior thermodynamic performance in comparison to other nanofluids, resulting in a 10% increase in heat exchange rate for the hydride reactor. The study found that the CuO/HTF nanofluid with a 5 vol% concentration performed better than the other nanofluids in the hydride reactor. This highlights the benefits of using nanofluids in this application.

The evaluation of various storage methods for green hydrogen reveals a diverse array of options, each with distinct advantages and challenges. Compressed hydrogen and liquid hydrogen offer straightforward and mature technologies but are hindered by high energy requirements and safety concerns related to pressurization and cryogenic temperatures. Chemical manufacturing of hydrogen carriers such as ammonia, methanol, and formic acid presents a promising alternative, providing a more stable and potentially safer means of storage and transportation. However, these methods require further optimization to improve the efficiency of hydrogen release and to reduce associated carbon emissions. On the other hand, metal hydrides, including sodium alanate and magnesium hydride, demonstrate significant potential due to their high hydrogen storage densities and relatively moderate operating conditions. Nevertheless, the commercialization of metal hydride storage is currently limited by issues related to material cost, kinetics, and cyclic stability.

It is clear that while several methods show promise, no single storage technology currently meets all the criteria for widespread adoption. Therefore, ongoing research is essential to address the technical and economic barriers associated with each storage method. Future studies should focus on enhancing the efficiency of hydrogen release, reducing material costs, and improving the safety and feasibility of large-scale deployment. By advancing these areas, the development of an optimal hydrogen storage solution can be accelerated, thereby facilitating the broader adoption of green hydrogen as a key component of the global energy transition.

## 4. Applications

The H_2_ economy is currently in existence, but it lacks environmental sustainability. Annually, the industrial sector is responsible for 6.3 billion metric tons of global energy-related carbon emissions.^132^ About 17% (1.1 billion metric tons) of carbon emissions come from the production of gray H_2_ feedstocks in chemical synthesis and industrial processes.^133^ It's essential to produce cheap and carbon free hydrogen.

### 4.1. Domestic uses

When considering the use of hydrogen for domestic energy, one significant challenge is its lower volumetric energy density compared to natural gas. Hydrogen's energy content per unit volume is considerably less than that of natural gas, necessitating a larger volume of hydrogen to achieve the same energy output. This disparity impacts the efficiency and practicality of using hydrogen in residential settings, as it would require more substantial storage infrastructure and potentially more frequent refilling or supply mechanisms to meet household energy demands. Additionally, the existing natural gas infrastructure is optimized for the higher energy density of natural gas, meaning adaptations or new infrastructure investments would be necessary to accommodate the higher volumes of hydrogen needed for equivalent energy provision. Addressing this issue is crucial for integrating hydrogen as a viable energy source in homes, ensuring that it can be supplied efficiently and economically.

Hydrogen has 2.4 times more energy per unit mass than methane. However, due to its low density, its lower heating value (LHV) per unit volume is three times lower than methane. This results in a reduction of the energy content in the gas blend as the hydrogen concentration increases. From a safety perspective, higher hydrogen concentrations raise the risk of fire and explosion. Hydrogen has a much broader flammability range (5.3 times) and detonation limit range (7.1 times) compared to methane. Additionally, it has a significantly lower ignition energy (14.5 times lower), making it more easily ignitable and increasing the fire risk.

Utilizing current pipeline systems to blend hydrogen with natural gas (Table 4) offers the most affordable means of transporting significant quantities of hydrogen over long distances without requiring new infrastructure. Nonetheless, because hydrogen molecules are smaller and have unique physical characteristics, including lower density and viscosity, the mixture exhibits behavior distinct from that of pure natural gas.^139,140^ This introduces potential safety hazards for pipelines designed specifically for natural gas. To keep the energy output consistent, the mixture with hydrogen may require higher flow rates, leading to increased operating pressures that could surpass the design limits of the compressors and pipelines originally meant for natural gas. Hence, it is essential to consider redesigning these systems to safely transport the hydrogen blend and to identify any risks and operational challenges associated with varying hydrogen concentrations. It is crucial to maintain a uniform mixture of the blended gas along the entire pipeline. Significant density differences between the gases can cause them to separate, leading to varied flow behaviors and leak issues. This separation can result in inconsistent energy distribution and operational challenges in the pipeline.^134^

**Table 4:** Hydrogen blending system^134–138^

Project	Country	Network	Electrolyser capacity	Hydrogen blend %
HyP SA	Australia	Distribution	1.2 MW	5%
ATCO-CEIH	Australia	Distribution	0.15 MW	5–25%
HyDeploy	UK	Distribution	0.5 MW	20%
Jupiter 1000	France	Transmission	1.0 MW	6%

M. Ozturk *et al.*,^142^ designed and studied an integrated system to produce renewable hydrogen and blend it with natural gas from the Black Sea for widespread use in Turkey. They focused on a case study for the city of Zonguldak, aiming to use the natural gas reserves more efficiently and environmentally. The study primarily investigates blending natural gas with 20% hydrogen by volume. Renewable energy sources such as wind, solar, and wave were evaluated for their hydrogen production capacities, yielding 1432 kg, 174 210 kg, and 1257 kg, respectively. The study examined the impact of this hydrogen addition on gas consumption and the lifespan of natural gas reserves. With 20% hydrogen, annual gas consumption increased from 46.55 billion cubic meters (bm^3^) to 54.11 bm^3^, while natural gas consumption decreased from 46.55 bm^3^ to 43.29 bm^3^, extending the reserve lifespan from 11.6 years to 12.5 years. Emissions of CO and CO_2_ dropped significantly, from 0.05 g day^−1^ and 32% to 0.02 g day^−1^ and 28%, respectively, as the hydrogen content increased from 5% to 20%. However, NO_*x*_ emissions rose from 4.08 g day^−1^ to 7.54 g day^−1^ with the same hydrogen increase.

**Fig. 7:**
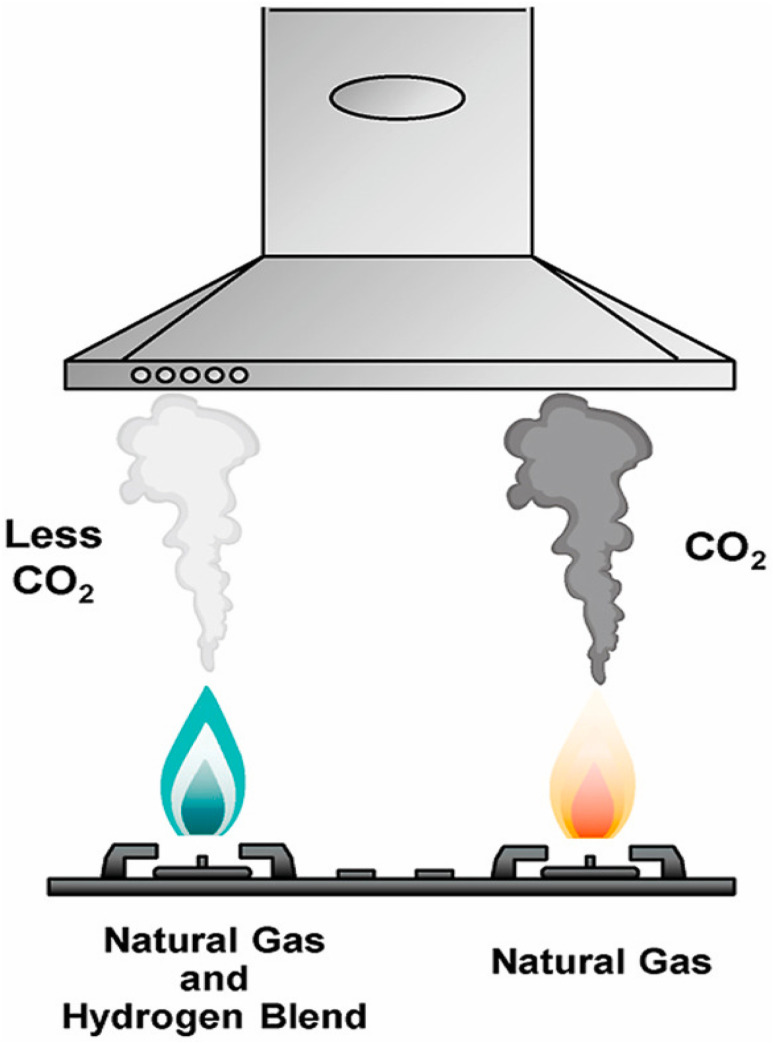
Illustration of the benefit from blending green H_2_ with natural gas.^141^

M. Ozturk *et al.* (Fig. 7),^141^ conducted an experimental investigation to analyze the impact of adding hydrogen to natural gas on emissions and combustion performance. They burned natural gas and various natural gas-hydrogen blends (with 10%, 20%, and 30% hydrogen by volume) in identical gas stoves and measured emissions of CO, CO_2_, and NO_*x*_. The results showed that increasing the hydrogen content improved combustion efficiency from 39.32% to 44.4%. Higher hydrogen ratios reduced CO_2_ and CO emissions, but NO_*x*_ emissions varied. A life cycle analysis assessed the environmental impact of the different blending scenarios. With a blend containing 30% hydrogen, the global warming potential decreased from 6.233 to 6.123 kg_CO_2_ equivalents_ per kg_blend_, and the acidification potential dropped from 0.0507 to 0.04928 kg_SO_2_ equivalents_ per kg blend compared to pure natural gas. However, there were slight increases in human toxicity, abiotic depletion, and ozone depletion potentials per kg blend, rising from 5.30 to 5.52 kg 1,4-dichlorobenzene (DCB) equivalents, 0.0000107 to 0.00005921 kg Sb equivalents, and 3.17 × 10^−8^ to 5.38 × 10^−8^ kg CFC-11 equivalents, respectively.

### 4.2. Steel manufacturing

The global steel industry currently uses approximately 5% of the world's energy and contributes over 6% of annual human-caused CO_2_ emissions.^143,144^ Approximately 15% of China's total CO_2_ emissions come from the steel industry.^145^ In China, more than 90% of crude steel is manufactured using the blast furnace-converter process, which relies heavily on coal-based energy sources. Due to the significant demand for steel products and its economic viability, this method is expected to remain the prevailing approach in the coming decades.^146^

In the steel industry, hydrogen serves two main purposes: (1) reducing iron oxide content in the blast furnace (BF) production process and the gas-based direct reduction iron (DRI) process, (2) it functions as a fuel for various heating applications, such as assisting in sintering production, the pelletizing process, and heating ladle furnaces, among others.^147,148^ Introducing hydrogen-enriched gases into the BF results in a reduction in the viscosity and density of the gas mixture. This reduction in density and viscosity leads to a lower pressure drop and faster heat exchange between the gas mixture and the materials being processed in the BF. As a result, this contributes to enhancing the efficiency of heat utilization in the BF.^147^ Simultaneously, during the reduction in iron oxides by hydrogen eqn (8), the diffusion capacity of hydrogen is 3.74 times greater than that of carbon monoxide. The internal micro and macro pores of the iron ores enable H_2_ to efficiently reach the reaction interface through diffusion.^149^ Consequently, if the H_2_/CO ratio is higher, the reduction rate will be faster with the same volume fraction of reduction agents. Numerous scholars have substantiated this through studies on the dynamic aspects of iron oxide reduction.^147,150–155^

8 Fe_2_O_3_ + 3H_2_ → 2Fe + 3H_2_O

It was clear now that hydrogen metallurgy offers several advantages. Firstly, it produces H_2_O as a reduction product, reducing reliance on fossil fuels like coal and coke and decreasing CO_2_ emissions. Additionally, H_2_ serves as a superior reductant compared to CO, thanks to its higher calorific value, lower density, enhanced penetration, and faster reduction rate. The availability of abundant raw materials for H_2_ production ensures a readily available supply. Moreover, H_2_ metallurgy can stimulate the rapid growth of DRI processes by substituting natural gas with H_2_, which is valuable in localities with limited natural gas resources, such as China. In general, H_2_ metallurgy plays a role in the sustainable development of iron and steel enterprises.

### 4.3. Chemical manufacturing (methanol, methane, green ammonia, formic acid)

**Fig. 8:**
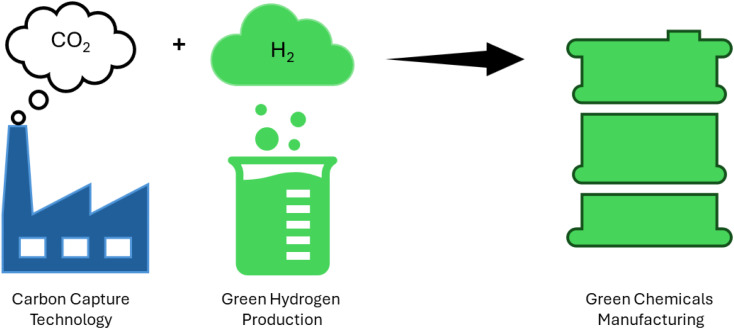
Illustration of the manufacturing of different organic materials using carbon capture and green hydrogen production.

**Fig. 9:**
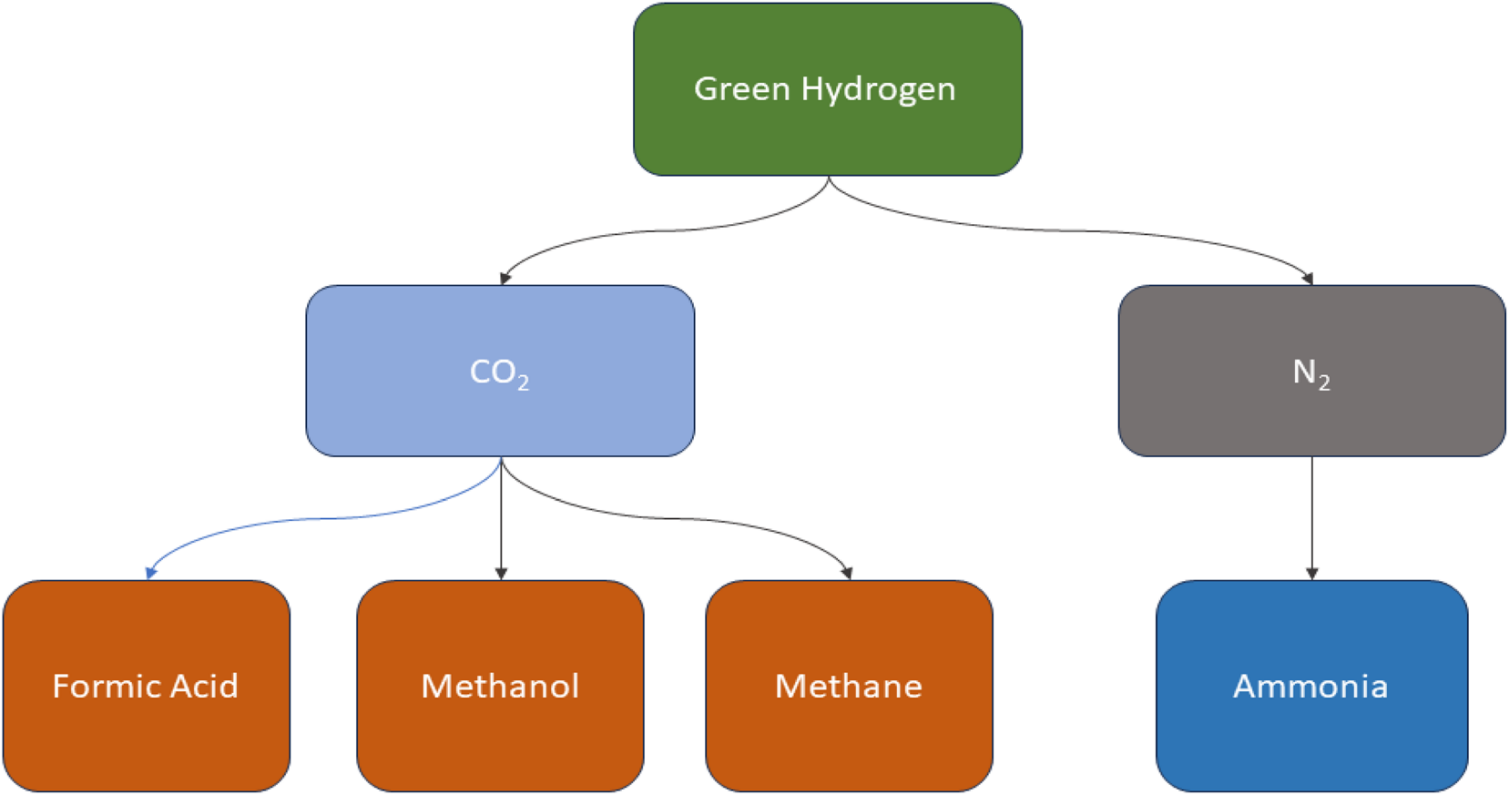
Showing the different chemical products that can be generated by green H_2_.

Conventional coal chemical production exhibits low energy efficiency and significant CO_2_ emissions. Coal has a hydrogen-to-carbon (H/C) ratio of approximately 4 : 5, while the resulting chemical products like natural gas, liquid fuels, olefins, and methanol have H/C ratios of around 3 : 1, 2 : 1, 2 : 1, and 2 : 1, respectively.^156,157^ The disparity in H_2_ and carbon content between chemical and coal products results in an excess of carbon and substantial CO_2_ emissions during coal conversion processes. Take the coal-to-methanol (CTM) conversion as an example, where the gas produced from coal gasification has an H/C ratio of around 0.7, while synthesized methanol has a ratio of about 2.0. The conversion of CO to H_2_ necessitates the water-gas shift reaction. During this process, the majority of the carbon is converted into CO_2_ and then emitted into the atmosphere. As a result, carbon resources are wasted and significant CO_2_ emissions are generated.^158,159^ Approximately 45% of energy is efficiently used in the CTM process, with CO_2_ emissions ranging from 3.8 to 4.3 metric tons per metric ton of methanol.^160,161^ As shown in (Fig. 8) green hydrogen is a great candidate for manufacturing various chemical compounds such as ammonia, methane, methanol, formic acid and so on without releasing carbon dioxide as in (Fig. 9).

#### 4.3.1. Methanol

**Fig. 10:**
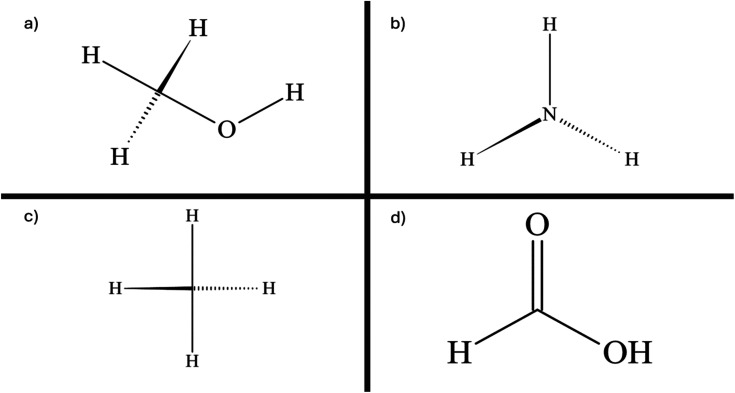
Visual representation of the chemical structure of (a) methanol, (b) ammonia, (c) methane, and (d) formic acid.

Methanol (Fig. 10a) is used as a crucial raw material in producing different chemicals, including methyl *tert*-butyl ether, acetic acid, dimethyl ether, formaldehyde, and various products like plastics, paints, construction materials, and automotive components.^162^ It is known for its versatility as a solvent and low-emission synthetic fuel used in various applications, such as wastewater treatment, cooking, industrial boilers, transportation, and electricity generation. Additionally, it serves as a carrier for chemical energy in fuel cells and an alternative medium for transporting H_2_.^162,163^ The traditional method involves fossil fuel-based synthesis gas, but it can also be produced by directly oxidizing natural gas or reducing atmospheric CO_2_ with H_2_. Producing green methanol by combining H_2_ and CO_2_ offers a way to reduce greenhouse gas emissions. This methanol can serve as both a sustainable transportation fuel and a means to store electricity.^164^

Dongliang and colleagues^165^ introduced an innovative approach for H_2_ production coupled with CO_2_ application in the coal-to-methanol (CTM) process. They termed this new approach the GH-CTM process, designed to enhance material integration, carbon efficiency, and methanol yield. Through comprehensive process modeling, parameter optimization, and simulations, the results demonstrated remarkable improvements compared to the conventional CTM process. The GH-CTM process exhibited a 10.52% higher energy efficiency, an 85.64% reduction in CO_2_ emissions, and a remarkable 124.67% increase in methanol production. In addition, the proposed process had significantly slowed production costs, 23.95% less than the traditional CTM process. Notably, the payback period for investment in the GH-CTM process was substantially shorter, at 2.8 years, compared to the CTM process's 7.2 years. Moreover, the GH-CTM process experienced a 47.37% increase in internal rate of return compared to the traditional CTM process. This new approach shows potential for introducing green H_2_, utilizing CO_2_, and transforming coal into valuable chemicals sustainably.

A preliminary assessment by Sollai and their team^166^ looked into a power-to-fuel plant setup for generating 500 kg h^−1^ of renewable methanol using green H_2_ and captured CO_2_. They developed a comprehensive process model employing the Aspen Plus tool, which simulated all aspects of the plant and the system as a whole. Once the process was optimized, a comprehensive economic analysis was performed, considering operating and capital costs derived from real-world experience at a commercial scale, with a projected operational lifetime of 20 years. Through the analysis, it was determined that the LCoM is 960 € per t, which translates to around 175 € per MW h. While the study showed that, as of the present, the technology isn't yet economically competitive, with the LCoM exceedingly double the prevailing international methanol price of 450 € per t, it does indicate a potential shift towards competitiveness in the medium-term future, largely driven by evolving European policies. Additionally, the research revealed that LCoM is particularly influenced by factors such as electricity prices, electrolyzer capital costs, and the plant's capacity factor.

#### 4.3.2. Green ammonia

Over the past ten years, global ammonia (Fig. 10b) production has experienced significant expansion, primarily driven by the top five nations, including Indonesia, the USA, India, Russia, and China, which collectively contribute to approximately 60% of the overall market. The exothermic reaction between nitrogen and hydrogen leads to ammonia synthesis, as shown in equation.^167^9
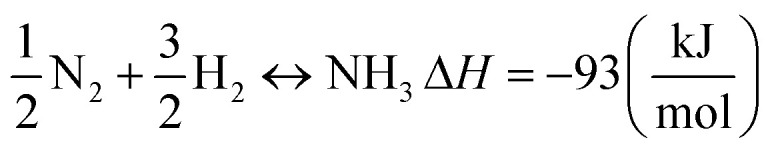


As outlined by MacFarlane *et al.*,^168^ various approaches for green ammonia production can be categorized: First-generation green ammonia involves capturing carbon emissions post-ammonia production and storing it, resulting in what is referred to as “blue ammonia”. Second-generation green ammonia focuses on producing ammonia from environmentally friendly feedstocks, namely N_2_ and H_2_. This approach aims to transform the traditional Haber–Bosch process into a sustainable source. Third-generation green ammonia entails departing from the conservative Haber–Bosch process and adopting alternative methods that prioritize high stability, sustainability, and the use of renewable sources for ammonia production.

Currently, various methods exist for the indirect generation of environmentally friendly ammonia, such as microbial electrolysis,^169^ photosynthesis,^170^ dark fermentation,^171^ and electrolysis.^172^ Electrochemical techniques have garnered significant attention in numerous nations.^173^

An enhanced optimization-based simulation model was introduced by Zhao and their team^174^ to simulate the long-term sustainability of green manufacturing. They investigated the effect of significant institutional incentives and the collaborative effects of O_2_ on investments. According to the study, the estimated levelized cost of ammonia is about 820 USD per t, which is nearly twice the current market price. Several factors were identified as pivotal in green ammonia investments, including the operational rate, the electrical efficiency of electrolyzers, electricity costs, and ammonia pricing. China's energy transition was greatly influenced by carbon pricing and VAT exemptions. To bridge the gap, a subsidy of about 450 USD per t would be needed based on the current pricing, but this could be lowered by 100 USD per t through the implementation of O_2_ synergy. Comparatively, green NH_3_ production exhibited both environmental and economic advantages when contrasted with inter-regional power transmission. The study thus advocates a balanced approach, leveraging both options to address integrating O_2_ manufacturing into H_2_ production and renewable power curtailment processes. By mitigating renewable power curtailment, this research aims to encouragement the increase of the H_2_ economy in China.

Ishaq and colleagues^175^ conducted a study to determine if offshore wind energy can be used to produce green ammonia and green H_2_. They used water and air as the main inputs. The green ammonia would then be transferred to onshore demand points using ships or pipelines. They performed a comprehensive year-long transient analysis to ensure the technology's reliability and adherence to quality standards before field deployment. Their approach involved integrating offshore wind energy with a water electrolysis unit and a seawater desalination system to generate renewable H_2_ for subsequent green ammonia synthesis. In terms of costs, the economic analysis revealed that the offshore wind farm and electrolyzer made up the majority, with 45% and 29% allocations, respectively. In order to meet the constant demand for green ammonia at a rate of 554 kg h^−1^, they concluded that an 80 000 kg H_2_ storage system was necessary, which could also provide ammonia to external customers. The study further illustrated the variations in ammonia production capacity over the course of the year, both with and without the H_2_ storage system. Importantly, it demonstrated that integrating a H_2_ storage system could ensure a steady supply of green ammonia throughout the year.

Bouaboula and the research team^176^ developed a new Techno-Economic (TE) modeling method. Their goal was to optimize the operation and design of a pilot-scale Green Ammonia plant. The intermittent nature of renewable energy sources is taken into account in this novel TE model. In order to deal with this, we examined multiple site locations that had consistent meteorological data each year. Furthermore, the model includes a unique Energy Management Strategy (EMS) to ensure a continuous power supply for the Haber–Bosch (HB) reactor. The EMS ensures the smooth distribution of power from renewable sources to charge and discharge Energy Storage Systems (ESS). Two main Key Performance Indicators (KPIs) were used to evaluating the plant's performance: Levelized Cost of Ammonia (LCOA) and HB Load Factor (LF). The findings indicated that the implemented EMS effectively reduced the fluctuations in RE sources by optimally distributing power across different time slots. Consequently, the HB LF rose by 56% to 65%, based on the particular RE setup. The increase in LF resulted in lower plant costs due to higher production yield outweighing investment and operational expenses. The PV/Battery scenario, consisting of 6 MW of PV and 11 MW h of battery capacity, was identified as the most efficient plant configuration, with a LCOA of $774 per t NH_3_. By 2050, the estimated cost of NH_3_ could decrease to $250 per ton according to a forecast. Furthermore, it suggests that green ammonia is expected to be economically competitive with conventional fossil fuel approaches by 2030.

#### 4.3.3. Green methane

Methane (Fig. 10c) (CH_4_), often referred to as synthetic natural gas (SNG), is a readily available fuel found in nature, being the primary component of natural gas. Despite its status as a significant greenhouse gas, methane finds utility in electricity generation and industrial chemical processes, where it's burned in steam generators and gas turbines. It also plays a substantial role in our daily lives, serving as fuel for ovens, water heaters, kilns, automobiles, and more.^177^ When compared to other hydrocarbon fuels, CH_4_ is known for its significantly lower CO_2_ emissions, making it a more environmentally friendly option.^178^

Pignataro *et al.*^179^ presented three management strategies (MSs) for controlling the H_2_ storage tank and methanation unit in the power-to-gas system. The most influential operational variables were determined through a systematic comparison of these MSs, and their impact on system performance was evaluated. The first strategy, denoted as MSA, stood out as the most straightforward of the three. When the produced H_2_ falls within the operational range, MSB closely resembled MSA in its behavior when operational constraints were breached. The control algorithm of MSC was similar to MSB, but the storage tank supplied different amounts of additional H_2_ during “in-range” methanation operations. While the methanation unit was running, we considered a scenario where the setpoint for methanation matched the flow rate from the electrolysis system (ES). The findings indicated that MSA and MSB exhibited similarities in the methanation unit and CH_4_ production utilization factor. Despite this, MSB demonstrated greater efficiency in handling methanation unit shutdowns, albeit with the drawback of needing a bigger storage system. On the other hand, MSC demonstrated the highest CH_4_ production but had more shutdowns and used a smaller storage system. Nonetheless, the results consistently showed a low average state of charge (SOC) for the storage in all MSs, suggesting that the system components may not have been sized optimally. Further investigation is needed to explore how resizing different subsystems impacts system performance and cost. Ultimately, the selection of the management strategy varies on the goal and feasibility of utilizing excess H_2_ in the power-to-methane system.

In a comprehensive study, Garcia-Luna *et al.*^180^ focused on integrating waste biomass oxycombustion with a power-to-methane system. Their approach primarily relies on using photovoltaic solar energy to drive PEM electrolysis and produce H_2_ and O_2_. The gases are utilized in a sub-critical steam power cycle for waste combustion. Depending on the operational strategy, an air separation process utilizing cryogenic distillation can generate an extra O_2_. Following purification and compression, the CO_2_ stream is directed towards the methanation reactor. The researchers created a quasi-stationary model to simulate the entire plant and assess integration efficiency under different operational conditions. According to their study, the entire plant integration shows high efficiency, with a CO_2_ reduction associated efficiency penalty of approximately 6% points on average per year. The system reduces emissions by using waste biomass as the primary fuel source, resulting in a −610 kg_CO_2__ per MW h reduction compared to biomass plants without CO_2_ capture. Furthermore, a comprehensive annual techno-economic study shows an average levelized electricity cost of €56 per MW h and an average green CH_4_ production cost of €12 per MW h. The results support the implementation of this system in both new and retrofitted biomass power plants because the CO_2_ capture cost is around 65.66 € per ton of CO_2_.

#### 4.3.4. Formic acid

Formic Acid (FA) (Fig. 10d) is a clear and intensely sharp-smelling liquid when maintained at standard room temperature and atmospheric pressure. It possesses the property of miscibility with water and a wide range of polar organic solvents, while showing partial miscibility with hydrocarbons. This versatility in solubility is attributable to its dual characteristics, featuring both acid and aldehyde functional groups, rendering it inherently reducing in nature.^181^ FA, recognized as a pivotal platform chemical, holds significant importance in both the chemical and agricultural sectors. In particular, it serves as a fundamental chemical compound extensively employed across various industries, including but not limited to chemical, textile, leather, pharmaceutical, and rubber industries.^182,183^ In the past decade, silage preservation and the use of formic acid as an additive in animal feed have outpaced its application in leather and tanning, making it the most significant global utilization of this compound.^183^

Gong and his team^184^ developed an advanced integrated system that merges methanol selective oxidation reaction and the H_2_ evolution reaction. By incorporating a power management system, this system operates on a UDC RF-Pulsed-TENG. At the cathode, green H_2_ is produced in this setup, while simultaneously generating the high-value chemical product, FA, at the anode. Applying a constant voltage of 1.8 V to the electrochemical cell after power management resulted in 1.68 times increase in the green H_2_ production rate. The entire system produces green H_2_ at a rate of 14.69 μL min^−1^ with 100% Faraday efficiency. Additionally, it allows for the simultaneous and quick generation of pure green H_2_ and valuable FA using clean energy sources.

### 4.4. Hydrogen fuel cells

Developed in 1839, fuel cells represent electrochemical devices with the remarkable ability to convert H_2_ and O_2_ directly into electricity, all while operating in an environmentally friendly manner, free from CO_2_ emissions. These devices present a compelling alternative, holding the promise of steering us towards a future of decarbonized energy solutions.^185^ Fuel cell technology consists of a trinity of key components: the cathode, the electrolyte, and the anode. Here, the anode plays a vital role, as H_2_ engages in an oxidation reaction (H_2_ → 2H^+^ + 2e^−^), setting free electrons in motion through an external circuit, while positively charged cations make their way towards the cathode by traversing the electrolyte. It's at the cathode that a critical reduction reaction unfolds (4H^+^ + O_2_ + 4e^−^ → 2H_2_O), involving both cations and electrons, ultimately converting O_2_ into water. These orchestrated chemical reactions are the cornerstone of fuel cell power generation.^186^ Cells that can serve as both fuel and electrolyzers cells within a single system are known as regenerative reversible or fuel cells. They derive this capability from their ability to reversibly split and recombine water (
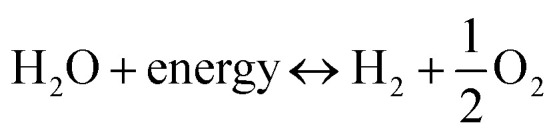
).^187^ Fuel cells offer a range of benefits, including adaptability, a modular structure, impressive energy density (ranging from 300 to 1200 Wh kg^−1^), strong power output, robust cycling performance, excellent thermal and mechanical stability, a prolonged lifespan (capable of enduring 20 000 or more discharge/charge cycles over 15 years), minimal vibration and noise, cost-effective maintenance, and straightforward installation and transportability.^188,189^ Nonetheless, the main hurdles for implementing this technology on a large scale revolve around the substantial upfront expenses and the complexities associated with widespread deployment.^190^ Different types such as molten carbonate fuels (MCFCs), phosphoric acid fuel cells (PAFCs), alkaline fuel cells (AFCs), proton exchange membrane fuel cells (PEMFCs), and solid oxide fuel cells (SOFCs) are compared in Table 5.^191^

**Table 5:** Comparison of different fuel cell types, these data taken from.^191^

	SOFCs	MCFCs	PAFCs	AFCs	PEMFCs
Electrolyte	Ceramics	Molten carbonate	Phosphoric acid	Potassium hydroxide	Polymeric membrane
Charge carriers	O^2−^	CO3^2−^	H^+^	OH^−^	H^+^
Operating temperature	500 to 1000 °C	600 to 700 °C	150 to 220 °C	50 to 200 °C	−40 to 120 °C (150 to180 °C in high temp. PEMFCs)
Electrical efficiency	Up to 65%	Up to 60%	Up to 45%	Up to 70%	Up to 65–72%
Primary fuel	H_2_, biogas, or methane	H_2_, biogas, or CH_4_	H_2_ or reformed H_2_	H_2_ or cracked ammonia	H_2_, reformed H_2_, methanol in direct methanol fuel cells
Primary applications	Stationary	Stationary	Stationary	Portable and stationary	Portable, transportation, and small-scale stationary
Power delivery (2019)	78.1 MW	10.2 MW	106.7 MW	0 MW	934.2 MW

## 5. Economics

The notion of a “H_2_ economy” was initially formulated by John Bockris during the 1970s. The idea is to produce H_2_ by electrolyzing water and then distribute it through pipelines to different locations. These locations would utilize on-site fuel cells to convert it back into electricity.^192^ Japan and other countries have detailed plans for a “H_2_ society”, where hydrogen is a key component of their energy systems, as stated in their Strategic Energy Plans.^193^ The “H_2_@Scale” vision has been introduced by scientists at the National Renewable Energy Laboratory. Various sectors, for instance industry, grid power, and transportation, incorporate H_2_ into their operations, resulting in advantages like increased energy security.^194^

The concept of the H_2_ economy is not far-fetched. Presently, gray and blue H_2_ are priced between $1.20 and $2.40 per kilogram, subject to the expense of carbon capture and storage. The cost of green hydrogen is approximately $4.85 per kilogram, taking into account an electricity cost of $53 per MW h and an efficiency of 65% at nominal capacity based on the lower heating value. However, it is anticipated that declining renewable electricity costs, enhanced electrolyzer efficiency, and reduced capital expenses will bring the cost of green H_2_ to below $2.00 per kilogram by 2030, making it competitive with gray H_2_ across various sectors, including industry.^195^ The refining sector is projected to see a rise in demand for H_2_ in the next decade, reaching approximately 41 million metric tons per year.^196^ Also, the demand for methanol and ammonia is projected to experience substantial growth in the foreseeable future, driven by their usage in agriculture and their role as efficient energy carriers.^197^

Several critical factors determine the economic viability of green H_2_ production plants. Firstly, it is essential to have a substantial H_2_ demand to justify the investment in such facilities. The industrial sector, particularly in applications such as steel production and chemicals, presents a significant opportunity for the utilization of green H_2_. Moreover, the economic feasibility is further enhanced when electricity prices are low, as the energy-intensive electrolysis process relies on affordable power sources. Additionally, the presence of high carbon taxes incentivizes industries to transition towards cleaner energy sources, making green H_2_ a cost-effective solution for reducing emissions.

However, there are numerous challenges and opportunities associated with green H_2_ production. One of the foremost challenges is the integration of these facilities with the existing energy grid. It can be challenging to balance intermittent renewable energy sources, such as solar and wind, with the steady demand for H_2_. Effective grid integration and energy storage solutions are critical to address this issue. Moreover, transportation and storage of H_2_, whether in gaseous or liquid form, pose logistical challenges. Infrastructure development is required to facilitate the efficient distribution and utilization of green hydrogen.

Lastly, policy support is paramount for the growth of the green H_2_ sector. Governments can incentivize investment through subsidies and regulations that promote cleaner energy sources. By addressing these challenges and capitalizing on the opportunities, green H_2_ production can become a transformative force in promoting sustainable energy solutions and reducing carbon emissions.

## 6. Limitations and future outlooks

It's evident that green H_2_ holds significant promise for transitioning to sustainable energy systems and substantially reducing carbon emissions. However, several challenges need to be addressed to realize its potential fully. The primary obstacle is the high production cost of green H_2_ compared to hydrogen derived from fossil fuels, driven by the expenses associated with renewable energy sources and electrolysis technology. Additionally, the intermittent nature of renewable energy sources like wind and solar impacts the continuous availability of green H_2_, making grid integration and energy storage solutions crucial for a stable supply. Efficient transportation and storage of hydrogen also pose challenges, requiring specialized infrastructure and incurring additional costs for processes like compression, liquefaction, or using chemical carriers. Furthermore, meeting the growing demand for green hydrogen through mass production is complex, necessitating the scaling up of production facilities while maintaining economic viability.

Despite these challenges, the outlook for green hydrogen integration into large-scale projects is promising, aligning with the vision for a sustainable future by 2050. Technological advancements and increased production scales are expected to lower the cost of green H_2_. As renewable energy becomes more competitive, the production costs of green H_2_ will benefit from reduced energy expenses. Research and development efforts are anticipated to yield more efficient and cost-effective electrolysis technologies, with breakthroughs in catalyst materials and cell designs significantly enhancing production efficiency. Establishing hydrogen infrastructure, such as pipelines and storage solutions, will facilitate the broad adoption of green H_2_ across various industries, including transportation and power generation. Supportive government policies, including subsidies and carbon pricing mechanisms, are increasingly recognizing the role of green H_2_ in decarbonizing industries, encouraging investment and growth in this sector. Regions rich in renewable energy are exploring the potential for exporting green H_2_, creating new economic opportunities and fostering international cooperation. Industries like chemicals and steel are progressively transitioning to green H_2_ as a cleaner feedstock, driving demand and further reducing costs through economies of scale.

## 7. Conclusion

The adaptability and environmental advantages of green hydrogen have led to increased attention in sustainable energy. Renewable sources like solar and wind energy power the electrolysis process that splits water, creating this environmentally friendly energy carrier. It represents hope in our transition to a low-carbon economy, unlike gray hydrogen, which is derived from fossil fuels and contributes to harmful emissions. Green hydrogen offers a cleaner and more sustainable alternative, supporting global efforts to fight climate change and reduce reliance on finite resources. The potential of green hydrogen lies in its ability to transform different sectors, particularly transportation and industry. Green hydrogen offers emission-free alternatives to conventional fossil fuel vehicles, making it a practical solution for governments and industries worldwide aiming to reduce transportation emissions. By replacing diesel and oil, it helps reduce harmful emissions and promotes energy independence and resilience in transportation. Moreover, green hydrogen has applications not only in transportation but also in chemical manufacturing and heavy industry. The versatility of this raw material opens up possibilities for sustainable production through different chemical processes. Green hydrogen enables the synthesis of chemicals like ammonia, methanol, and ethanol, reducing carbon emissions and fostering innovation and competitiveness in these industries. Green hydrogen holds the potential for cost and energy reduction in steelmaking. Replacing coke and coal with hydrogen in iron reduction can greatly reduce greenhouse gas emissions, promoting sustainability in heavy industries. The rise of green hydrogen signifies a major transition to a greener, more sustainable energy future. Its significance in our joint endeavors to combat climate change and build a sustainable future for generations to come is highlighted by its ability to reduce carbon emissions in transportation, transform chemical manufacturing, and enhance the efficiency of heavy industries.

## Data availability

No new data were created or analysed during this study. Data sharing is not applicable to this article.

## Conflicts of interest

There are no conflicts to declare.
